# Correlation analysis between short-term insulin-like growth factor-I and glucose intolerance status after transsphenoidal adenomectomy in acromegalic patients: a large retrospective study from a single center in China

**DOI:** 10.20945/2359-3997000000118

**Published:** 2019-03-18

**Authors:** Yi-Lin Li, Shuo Zhang, Xiao-Peng Guo, Lu Gao, Wei Lian, Yong Yao, Kan Deng, Ren-Zhi Wang, Bing Xing

**Affiliations:** 1 Peking Union Medical College Hospital Department of Neurosurgery Peking China Department of Neurosurgery, Peking Union Medical College Hospital, Peking, China; 2 Chinese Academy of Medical Sciences Plastic Surgery Hospital Department No. 16 Beijing China Department No. 16, Plastic Surgery Hospital, Chinese Academy of Medical Sciences, Beijing, China; 3 The Ministry of Health Key Laboratory of Endocrinology Peking China The Ministry of Health Key Laboratory of Endocrinology, Peking, China

**Keywords:** Glucose tolerance, diabetes, acromegaly, transsphenoidal surgery, insulin-like growth factor-I

## Abstract

**Objectives:**

Our study aimed to investigate the associations of glucose tolerance status with insulin-like growth factor-I (IGF-I) and other clinical laboratory parameters of acromegalic patients before and after the patients underwent transsphenoidal adenomectomy (TSA) by conducting a single-center, retrospective study.

**Subjects and methods:**

A total of 218 patients with acromegaly who had undergone TSA as the first treatment were retrospectively analyzed. Serum IGF-I, growth hormone (GH) and glucose levels were measured before and after surgery.

**Results:**

The follow-up levels for random GH, GH nadir, and the percentage of the upper limit of normal IGF-I (%ULN IGF-I) were decreased significantly. The percentages of normal (39.0%), early carbohydrate metabolism disorders (33.0%) and diabetes mellitus (28.0%) changed to 70.2%, 16.5% and 13.3%, respectively, after TSA. %ULN IGF-I at baseline was higher in the diabetes mellitus (DM) group than in the normal glucose tolerance group and impaired glucose tolerance (IGT) /impaired fasting glucose (IFG) groups before TSA, and the DM group exhibited a greater reduction in %ULN IGF-I value after surgery. The follow-up %ULN IGF-I value after surgery was significantly lower in the improved group, and Pearson's correlation analysis revealed that the reductions in %ULN IGF-I corresponded with the reductions in glucose level.

**Conclusion:**

This study examined the largest reported sample with complete preoperative and follow-up data. The results suggest that the age- and sex-adjusted IGF-I level, which reflects altered glucose metabolism, and the change of it are associated with improved glucose tolerance in acromegalic patients both before and after TSA.

## INTRODUCTION

Acromegaly results from the persistent hypersecretion of growth hormone (GH) and insulin-like growth factor-I (IGF-I), which causes most of the clinical manifestations of acromegaly. The etiology of more than 95% of acromegaly cases is a GH-secreting pituitary adenoma (1). Uncontrolled acromegaly is usually associated with a series of glucose metabolic disorders (2,3). Diabetes and other forms of impaired glucose metabolism with acromegaly are associated with an increased rate of mortality by promoting atherosclerosis, which results in cardiovascular and cerebrovascular diseases (4). Selective transsphenoidal surgical resection, which is one of the most important treatment options for patients with GH-secreting pituitary adenoma (5), can reduce GH levels and improve disordered metabolic functions (1).

Although several studies have investigated the relationship between IGF-I level and insulin resistance, few studies have investigated the correlations between IGF-I or other clinical laboratory parameters and glucose tolerance status, and none have reported a positive correlation between IGF-I and glucose tolerance status both before and after transsphenoidal adenomectomy (TSA) in a large sample with complete follow-up data (6–10). Therefore, we analyzed 218 acromegalic patients who had undergone TSA for acromegaly at our hospital to investigate the associations between glucose tolerance status and both IGF-I and other clinical laboratory parameters before and after TSA.

## SUBJECTS AND METHODS

### Subjects

In this study, we analyzed 345 consecutive patients who had been diagnosed with acromegaly caused by a GH-secreting pituitary adenoma without dysfunctions of other endocrine axes and who had undergone TSA at Peking Union Medical College Hospital (Beijing, China) between July 1, 2012 and December 31, 2014. Data regarding routine medical procedures were collected retrospectively. All 345 patients underwent TSA via the same procedure, and the diagnosis of each patient was confirmed by experienced pathologists who analyzed the tumor tissue after surgery.

Among the 345 patients, 74 received medical treatment for acromegaly and 21 received gamma-knife radiosurgery before surgery. A total of 250 patients received TSA as the first treatment. The tumors in 5 of these patients were not completely removed during surgery, and 27 patients were lost to follow-up. The remaining 218 patients who underwent surgery that was performed by surgeons with equivalent surgical experience were selected for data analysis. The baseline characteristics of the patients (*n* = 218) stratified by preoperative glucose tolerance status, i.e., normal glucose tolerance (NGT) (*n* = 85), IGT/IFG (*n* = 72), and diabetes mellitus (DM) (*n* = 61), are shown in Table 1. The mean disease duration time of these patients was 74.91 ± 65.48 months, and the mean ± SD age, BMI, systolic blood pressure (SBP), and DBP were 40.7 ± 12.8 years, 26.36 ± 4.52 kg/m^2^, 124.06 ± 16.53 mmHg, and 77.06 ± 12.35 mmHg. Women composed 58.7% of the subjects. The follow-up interval of the patients was defined as the time interval between surgery and the first follow-up date, and the mean follow-up interval was 127.23 ± 71.04 days.

**Table 1:** Preoperative clinical characteristics of the acromegalic patients

	Total (*n* = 218)	NGT (*n* = 85)	IGT/IFG (*n* = 72)	DM (*n* = 61)	P
Female, n (%)	128 (58.7)	50 (58.8)	38 (52.8)	40 (65.6)	0.328
Age, years	40.65 ± 12.83	37.91 ± 12.64	38.22 ± 11.24	47.34 ± 12.61	0.000
BMI, kg/m^2^	26.36 ± 4.52	25.74 ± 4.27	27.00 ± 5.45	26.46 ± 3.50	0.217
DD, months	74.91 ± 65.48	76.35 ± 63.17	70.94 ± 67.50	77.59 ± 67.09	0.817
SBP, mmHg	124.06 ± 16.53	120.85 ± 14.34	124.61 ± 15.98	127.87 ± 19.21	0.038
DBP, mmHg	77.06 ± 12.35	76.01 ± 10.73	76.13 ± 14.14	79.61 ± 12.04	0.164
Follow-up interval, days	127.23 ± 71.04	116.93 ± 63.81	130.67 ± 66.31	137.54 ± 84.13	0.198

The data are presented as absolute numbers (n) and percentages (%) or as the mean ± SD. SBP: systolic blood pressure; DBP: diastolic blood pressure; DD: disease duration; NGT: normal glucose tolerance; IGT/IFG: early carbohydrate metabolism disorders; DM: diabetes mellitus.

The diagnosis of acromegaly was based on failure of serum GH suppression to < 0.4 μg/L during the 75-g oral glucose tolerance test (OGTT) and an elevated serum IGF-I level. Pituitary adenoma was confirmed by magnetic resonance imaging (MRI) and by clinical features, including acral enlargement, increased skin thickness, increased sweating, DM, hypertension, headache, sleep apnea and osteopenia. Glucose tolerance status was evaluated by the 75-g OGTT. According to WHO criteria (11), NGT was defined as a blood glucose level < 110 mg/dL before glucose loading and < 140 mg/dL 2h after. DM was defined as a fasting blood glucose level of ≥ 126 mg/dL or a blood glucose level of ≥ 200 mg/dL at 2h after glucose loading. The remaining patients received a diagnosis of an early carbohydrate metabolism disorder, including IGT or IFG.

### Biochemical measurements

All of the serum samples were collected early in the morning after an eight-hour fasting period. The serum basal GH concentration was defined as the fasting GH concentration prior to administration of the 75-g OGTT. GH nadir (GHn) values were obtained during the 75-g OGTT, which was performed after a 12h fast. Blood samples were drawn to assess the baseline GH, IGF-I and glucose levels, with GH and glucose being assessed at 30, 60, 120 and 180 min. The follow-up 75-g OGTT was performed 127.23 ± 71.04 days after surgery, at the first follow-up. It is strongly recommended that IGF-I assays be calibrated using the WHO international standard of highly purified recombinant IGF-I (WHO IS 02/254). Furthermore, the GH assay should be calibrated to the 22-kDA isoform standard or, as a second choice, to multiple isoforms (12). Accordingly, GH levels were measured using an IMMULITE 2000 automated chemiluminescence analyzer (L2KGRH2, Siemens Healthcare Diagnostics Products Ltd., Glyn Rhonwy, Llanberis, Gwynedd LL55 4EL, UK) after an eight-hour fasting period and administration of the 75-g OGTT. The IGF-I levels were measured using an IMMULITE 2000 chemiluminescence analyzer (L2KGFZ, Siemens Healthcare Diagnostics Products Ltd., Glyn Rhonwy, Llanberis, Gwynedd LL55 4EL, UK) and were compared to levels in control individuals of the same age and gender. The IGF-I level is expressed as a percentage of the upper limit of the normal IGF-I (%ULN IGF-I) level for age and sex for each individual laboratory sample because the IGF-I SDS scoring system has not been applied to the evaluation of IGF-I in our hospital and because one of the latest guidelines does not use IGF-I SDS as the diagnostic standard (13).

### Criteria for a biochemical cure

The common consensus criteria are an IGF-I level in the age-adjusted normal range and a GH level < 1.0 μg/L from a random GH measurement, with nadir GH levels < 0.4 μg/L in patients undergoing neurosurgery (12).

### Statistical analysis

All statistical analyses were performed using IBM SPSS 20.0 software (SPSS Inc., Chicago, IL, USA). The data are reported as the mean ± SD for normally distributed continuous variables and as the number and percentage for dichotomous variables. The IGF-I level was calculated and expressed as a percentage of the upper limit of the normal IGF-I (%ULN IGF-I) level for each subject according to age using the Peking Union Medical College Hospital laboratory samples (%ULN=(IGF-I-ULN)/ULN×100%). Data were compared between groups using the χ^2^ test for categorical data and the t-test or ANOVA for continuous data. Associations between the two clinical laboratory parameters were assessed by Pearson's correlation coefficient analysis. A two-tailed *p* < 0.05 was considered to indicate statistically significant differences.

## RESULTS

### Clinical characteristics of the subjects before surgery

Our study showed that 28.0% of the patients had diabetes and 33.0% had IFG or IGT, with the remaining 39.0% of patients having normal glucose tolerance. The mean follow-up interval was 127 ± 71 days, with no significant difference between groups. There were gradual increases in age and SBP from NGT to IGT/IFG to DM. The patient age was higher in the DM group than in the NGT (*p* = 0.000) and IGT/IFG (*p* = 0.000) groups, but it did not differ between the NGT and IGT/IFG groups (*p* = 0.871). The SBP was higher in the DM group than in the NGT group (*p* = 0.011).

The preoperative clinical laboratory parameters are shown in Table 2. There were significant differences in the %ULN IGF-I level before surgery among the three groups (*p* = 0.005); this level was higher in the DM group than in the NGT (*p* = 0.001) and IGT/IFG (*p* = 0.028) groups, but it did not differ between the NGT and IGT groups (*p* = 0.325). The GH and GHn levels did not differ significantly among the groups.

**Table 2:** Clinical laboratory parameters of patients after surgery at follow-up

		Total (*n* = 218)	NGT (*n* = 85)	IGT/IFG (*n* = 72)	DM (*n* = 61)	p
Random GH < 1.0 μg/L, *n* (%)		116 (53.2)	46 (54.1)	36 (50.0)	34 (55.7)	0.786
GHn < 0.4 μg/L, *n* (%)		124 (56.9)	45 (52.9)	40 (55.6)	39 (63.9)	0.401
Normal age-adjusted IGF-I, *n* (%)		73 (33.5)	30 (35.3)	21 (29.2)	22 (36.1)	0.634
Both GHn < 0.4 μg/L and normal age-adjusted IGF-I, *n* (%)		67 (30.7)	28 (32.9)	18 (25.0)	21 (34.4)	0.428
Glu0h, mg/dL						
	At baseline	113.14 ± 32.79	96.57 ± 7.93	105.39 ± 10.09	145.20 ± 46.66	0.000
	At follow-up	101.61 ± 17.30	94.04 ± 8.83	97.47 ± 10.99	116.92 ± 21.98	0.000
	(p*)	(0.000)	(0.051)	(0.000)	(0.000)	
	Change from baseline	-11.53 ± 25.22	-2.52 ± 9.37	-7.93 ± 9.91	-28.29 ± 40.72	0.000
Glu2h, mg/dL						
	At baseline	168.27 ± 77.29	107.56 ± 18.92	156.56 ± 24.86	266.82 ± 71.34	0.000
	At follow-up	123.41 ± 59.99	98.73 ± 22.52	106.47 ± 32.25	177.64 ± 82.87	0.000
	(p*)	(0.000)	(0.007)	(0.000)	(0.000)	
	Change from baseline	-44.86 ± 54.95	-8.64 ± 25.76	-50.08 ± 32.97	-89.18±69.72	0.000
Random GH, μg/L						
	At baseline	32.68 ± 44.92	35.93 ± 53.37	31.23 ± 42.70	29.87 ± 33.56	0.686
	At follow-up	5.00 ± 8.98	5.74 ± 9.32	4.29 ± 5.69	4.82 ± 11.44	0.584
	(p*)	(0.000)	(0.000)	(0.000)	(0.000)	
	Change from baseline	-27.68 ± 43.01	-30.19 ± 51.17	-26.94 ± 41.54	-25.06 ± 31.11	0.766
GHn, μg/L						
	At baseline	24.21±36.65	27.81 ± 45.94	22.06 ± 31.95	21.73 ± 25.77	0.512
	At follow-up	2.94 ± 5.67	3.61 ± 6.42	2.44 ± 4.38	2.60 ± 5.90	0.377
	(p*)	(0.000)	(0.000)	(0.000)	(0.000)	
	Change from baseline	-21.27 ± 35.04	-24.21 ± 44.27	-19.62 ± 30.73	-19.14 ± 23.67	0.614
%ULN IGF-I						
	At baseline	200.95 ± 112.76	178.4 ± 101.83	195.88 ± 101.66	238.38 ± 130.62	0.005
	At follow-up	59.71 ± 91.45	68.42 ± 102.93	61.51 ± 88.94	45.44 ± 75.72	0.321
	(p*)	(0.000)	(0.000)	(0.000)	(0.000)	
	Change from baseline	-141.22 ± 120.43	-109.89 ± 111.34	-134.31 ± 111.58	-193.05 ± 127.22	0.000

Glu0h: basal glucose level on OGTT; Glu2h: 2-h glucose level on OGTT; GH: growth hormone; GHn: GH nadir; IGF-I: insulin-like growth factor-I; ULN: upper limit of normal range.

*p: p* value among the three groups (NGT, IGT/IFG, and DM) as determined by ANOVA. (P*): *p* value between preoperative and follow-up parameters; change from baseline = follow-up-baseline.

### Associations of clinical parameters with glucose tolerance after TSA

After TSA, the average Glu0h (basal glucose level on OGTT) and Glu2h (2-h glucose level on OGTT) decreased significantly in the IGT/IFG and DM groups (Table 2). The change from the baseline Glu0h was significantly higher in the DM group than in the IGT/IFG (*p* = 0.000) and NGT groups (*p* = 0.000), but there was no significant difference between the IGT/IFG and NGT groups (*p* = 0.145). The change from the baseline Glu2h was significantly different among the three groups (*p* = 0.000). The Glu2h of the DM group decreased the most, followed by the IGT/IFG group, and the differences between any two of the three groups were significant (*p* = 0.000).

As shown in Table 2, compared to the baseline levels, the follow-up random GH, GHn, and %ULN IGF-I levels decreased significantly in all three groups (*p* < 0.01). The changes from the baseline GH and GHn levels did not differ significantly among the groups. However, the change from the baseline %ULN IGF-I of the DM group was higher than were those of the IGT/IFG (*p* = 0.004) and NGT groups (*p* = 0.000), whereas it did not significantly differ (*p* = 0.191) between the IGT/IFG and NGT groups. According to the remission criteria, 53.3% (116 of 218) of the patients achieved a random GH level of < 1.0 μg/L, 56.9% (124 of 218) achieved a GHn level of < 0.4 μg/L, 33.5% (73 of 218) achieved a normal age-adjusted IGF-I level, and 30.7% (67 of 218) achieved both a GHn level of < 0.4 μg/L and a normal age-adjusted IGF-I level. We compared the percentage of patients with a random GH level of < 1.0 μg/L, GHn level of < 0.4 μg/L, and normal age-adjusted IGF-I level among the three groups after surgery. However, no significant differences were observed.

Logistic regression analysis including age, BMI, SBP, follow-up Glu0h, follow-up Glu2h, and the parameters random GH, GHn and %ULN IGF-I was carried out to identify parameters associated with preoperative glucose status. The baseline %ULN IGF-I (*p* = 0.020, OR = 1.006) and change from the baseline %ULN IGF-I (*p* = 0.000, OR = 1.105) were the variables most significantly correlated with preoperative glucose status for these patients.

### Association of clinical parameters with improved glucose tolerance after TSA

**Figure 1:**
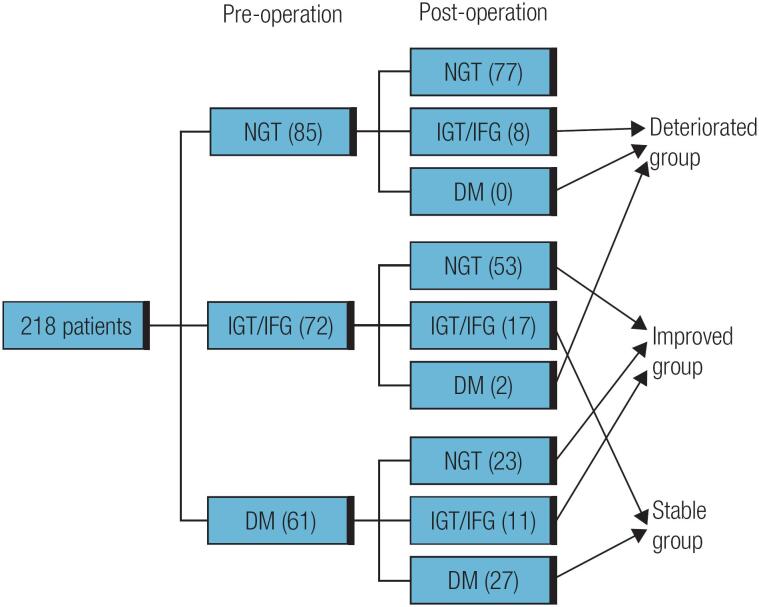
Categorization of the patients into three groups according to their preoperative and postoperative glucose tolerance statuses.

To determine how the clinical parameters are related to preoperative glucose tolerance status after surgery, the patients with abnormal glucose tolerance (IGT/IFG and DM) were divided into the following three groups according to the preoperative and postoperative glucose tolerance status (Figure 1): an improved group (*n* = 44), including the patients with IGT/IFG before surgery who experienced restoration of NGT after surgery and those with DM before surgery but with IGT/IFG or NGT after surgery; a stable group (*n* = 87), including the patients with persistent IGT/IFG or DM before and after surgery; and a deteriorated group (*n* = 10), including the patients with IGT/IFG before surgery who developed DM after surgery. Because the number of patients in the deteriorated group was too low to generate accurate statistics, this group of patients was not included in the comparative analysis. The glucose status changes in the three groups after surgery are shown in Figure 1. Surgery improved glucose tolerance in 54.1% (33 of 61) of the patients in the DM group and in 73.6% (53 of 72) of those in the IGT/IFG group.

As shown in Table 3, the percentage of females in the improved group was significantly lower than that in the stable group (*p* = 0.021), whereas the average age in the improved group was significantly higher than that in the stable group (*p* = 0.013).

**Table 3:** Clinical parameters of patients according to glucose tolerance before and after surgery

		Stable (*n* = 44)	Improved (*n* = 87)	p
Female, *n* (%)		32 (72.7)	45 (51.7)	0.021
Age, years		46.14 ± 11.86	40.31 ± 12.76	0.013
BMI, kg/m^2^		26.66 ± 3.10	26.79 ± 5.10	0.853
DD, months		85.36 ± 85.16	68.29 ± 56.03	0.233
SBP, mmHg		127.23 ± 19.32	125.71 ± 16.83	0.644
DBP, mmHg		80.20 ± 13.33	76.48 ± 13.31	0.133
Follow-up interval, days		133.16 ± 72.57	134.06 ± 76.89	76.89
Random GH < 1.0 μg/L, *n* (%)		17 (38.6)	51 (58.6)	0.031
GHn < 0.4 μg/L, *n* (%)		21 (47.7)	56 (64.4)	0.068
Normal age-adjusted IGF-I, *n* (%)		9 (20.5)	34 (39.1)	0.032
Glu0h, mg/dL				
	At baseline	148.27 ± 52.79	111.52 ± 19.10	0.000
	At follow-up	124.13 ± 21.98	97.29 ± 9.55	0.000
	(p*)	(0.007)	(0.000)	
	Change from baseline	-24.14 ± 45.94	-14.05 ± 17.30	0.074
Glu2h, mg/dL				
	At baseline	251.14 ± 92.06	185.56 ± 54.77	0.000
	At follow-up	204.84 ± 78.55	105.21 ± 32.61	0.000
	(p*)	(0.013)	(0.000)	
	Change from baseline	-46.30 ± 54.05	-80.35 ± 53.33	0.001
Random GH, μg/L				
	At baseline	31.18 ± 35.16	30.86 ± 40.74	0.965
	At follow-up	5.29 ± 6.57	4.23 ± 9.79	0.519
	(p*)	(0.000)	(0.000)	
	Change from baseline	-25.89 ± 33.24	-26.63 ± 39.23	0.915
GHn, μg/L				
	At baseline	22.31 ± 26.37	22.13 ± 30.83	0.974
	At follow-up	3.39 ± 5.14	2.12 ± 5.12	0.182
	(p*)	(0.000)	(0.000)	
	Change from baseline	-18.92 ± 24.75	-20.02 ± 29.28	0.832
%ULN IGF-I				
	At baseline	238.34 ± 126.49	204.71 ± 111.15	0.121
	At follow-up	95.20 ± 97.28	35.94 ± 70.19	0.001
	(p*)	(0.000)	(0.000)	
	Change from baseline	-143.09 ± 140.36	-168.84 ± 111.57	0.256

The baseline and follow-up Glu0h and Glu2h of the improved group were significantly lower than those of the stable group (*p* < 0.01). The change from the baseline Glu2h in the improved group was significantly higher than that in the stable group (*p* = 0.001); the mean change from the baseline Glu0h was also higher in the improved group, but this difference was not statistically significant (*p* = 0.074).

The baseline, follow-up, and change from the baseline levels of random GH and GHn were similar in both groups. However, the follow-up %ULN IGF-I after surgery was significantly lower in the improved group than in the stable group (*p* = 0.001), whereas the baseline and change from the baseline %ULN IGF-I did not significantly differ. Moreover, the improved group had a significantly higher proportion of patients with a random GH level of < 1.0 μg/L (*p* = 0.031) and normal age-adjusted IGF-I level (*p* = 0.032) than the stable group. In addition, the improved group had an increased proportion of patients with a GHn level of < 0.4 μg/L (*p* = 0.068) that approached statistical significance.

Logistic regression analysis of age, BMI, and the parameters random GH, GHn, and %ULN IGF-I showed that an improvement in glucose tolerance status was associated with a lower follow-up %ULN IGF-I (*p* = 0.001, OR = 1.011) and could be predicted by younger age (*p* = 0.044, OR = 1.042). According to the ROC curve analysis, the age cut-off value for predicting an improvement in glucose tolerance status after TSA was 45.5. The sensitivity and specificity were 59.1% and 29.9%, respectively, and the AUC was 63.3% (53.4%-73.2%) (*p* = 0.013).

### Comparisons of glucose levels according to IGF-I criterion of a cure

To suppress the interference of GH, we divided the 218 patients into cure (*n* = 73) and discordant (*n* = 145) groups according to the IGF-I criterion of a cure (a normal age-adjusted IGF-I) to compare the glucose levels between the two groups (Table 4).

**Table 4:** Comparisons of glucose levels of the cure and discordant groups according to the IGF-I criterion of a cure

		Cured (*n* = 73)	Discordant (*n* = 145)	p*
Follow-up interval, days		130.23 ± 75.32	125.72 ± 69.01	0.659
Glu0h, mg/dL				
	At baseline	109.36 ± 22.88	114.94 ± 36.75	0.225
	At follow-up	97.11 ± 14.41	103.77 ± 18.20	0.007
	Change from the baseline	-12.07 ± 17.84	-11.17 ± 28.29	0.805
Glu2h, mg/dL				
	At baseline	165.39 ± 70.98	169.71 ± 80.53	0.706
	At follow-up	112.96 ± 56.03	128.63 ± 61.43	0.070
	Change from the baseline	-52.43 ± 56.21	-41.08 ± 54.23	0.149

The data showed that the follow-up Glu0h of the cure group was significantly lower than that of the discordant group (*p* = 0.007), and the average follow-up Glu2h was also lower in the cure group, approaching significance (*p* = 0.070).

### Clinical laboratory parameters and their associations with glucose tolerance

We analyzed the relationships among the %ULN IGF-I and GH levels and glucose parameters. The %ULN IGF-I before surgery was positively correlated with all parameters before surgery, including the GH, GHn, Glu0 and Glu120 levels (rGH = 0.193, rGHn = 0.184, rGlu0 = 0.180, and rGlu120 = 0.231, *p* < 0.01). Reductions in the %ULN IGF-I corresponded with reductions in the glucose level (rGlu0 = 0.198 and rGlu120 = 0.168, *p* < 0.05). The changes in the %ULN IGF-I before and after surgery were correlated positively with changes in the glucose level (rΔGlu0 = 0.220, rΔGlu120 = 0.297, *p* < 0.01). The GH-related indexes were not significantly correlated with any of the glucose parameters.

## DISCUSSION

Glucose tolerance is frequently altered in acromegaly. IGT and overt diabetes are usually associated with the acromegalic condition, and their prevalence rates range from 16 to 46% and from 19 to 56%, respectively (14).

The effects of GH and IGF-I on glucose metabolism are very complex. GH hypersecretion leads to increases in both hepatic (increased gluconeogenesis in hepatocytes) and peripheral insulin resistance in adipose tissue and muscles (15,16). In contrast, IGF-I increases insulin sensitivity and lowers the blood glucose level. IGF-I may indirectly regulate carbohydrate metabolism through both the suppression of GH and enhancement of insulin activity (17). IGF-I reduces the serum GH concentration and GH-related insulin suppression of hepatic gluconeogenesis by increasing free fatty acid uptake in muscle, which indirectly enhances the activity of hepatic insulin (18). In addition, IGF-I directly stimulates glucose transport into muscle through either IGF-I or insulin/IGF-I hybrid receptors (19,20). However, in acromegalic patients, the negative effects of GH largely overwhelm the possible beneficial effects of IGF-I on insulin sensitivity. Studies have reported that the plasma GH and IGF-I levels in acromegaly are associated with the insulin resistance status (21). In our study, in agreement with this finding, baseline %ULN IGF-I was significantly associated with the worse glucose status before surgery, whereas plasma GH level was not.

We also found significant decreases in the levels of biochemical parameters, including Glu0h, Glu2h, random GH, GHn, and %ULN IGF-I, in our patients after surgery. These results suggest that TSA can indeed improve the glucose status in acromegaly, which should be analyzed in a large single-center sample. In accordance with previous observations (10,22,23), we observed that age and hypertension were significantly associated with the presence of diabetes in this sample; however, unexpectedly, no significant associations were found for sex, BMI, or disease duration.

According to the χ^2^ test, the proportions of biochemically cured patients did not significantly differ among the three groups, but they were slightly higher in the DM group than in the other two groups. In addition, we found that the patients with preoperative DM had significantly greater decreases in Glu0h, Glu2h, and %ULN IGF-I after surgery than in the IGT/IFG and NGT groups (Table 2). These results may be due to the initially higher %ULN IGF-I levels in the DM group; however, the average follow-up %ULN IGF-I was the lowest in that group, although this latter difference was not significant. This suggests that DM patients with pituitary GH adenoma might be more likely to benefit from the operation due not only to a greater decrease in %ULN IGF-I but also to a greater improvement in blood glucose level. The concentration of IGFBP-3, the main IGF carrier protein, is usually increased in acromegalic patients (24), and studies have indicated that the concentration of this protein is positively correlated with that of IGF-I (25). Therefore, the higher %ULN IGF-I before surgery in the DM group likely led to a higher IGFBP-3 level in this group, potentially increasing the binding of IGF-I and reducing the IGF-I level in the blood shortly after surgery. Such a mechanism might partially explain why the DM group had a lower %ULN IGF-I at follow-up. In addition, IGF-I is credited with reducing insulin resistance and the blood glucose level (17). One possible explanation for the greater decrease in the glucose level in the DM group is that diabetes patients with a high insulin level can have increased sensitivity of IGF-I receptors (26). Similar to patients with glucose metabolic disorder that is caused only by high GH levels in the blood, the reduction or elimination of GH level can make such patients exhibit enhanced reactivity to IGF-I.

We analyzed the clinical parameters associated with the improved glucose tolerance status in this sample. The glucose tolerance of the IGT/IFG group was more likely to be improved than that of the DM group. In accordance with this expectation, the improved group had better glucose tolerance and more easily achieved a biochemical cure than the stable group, especially with regard to the random GH and normal age-adjusted IGF-I levels. The results also revealed that the patients in the improved group were younger, that fewer were female, and that they had a lower %ULN IGF-I at follow-up. As age is one of the risk factors for decreased glucose tolerance in acromegaly (27), younger patients may have a greater possibility of improving their glucose status through operative treatment.

Similar to previous studies, the IGF-I levels fluctuated during the early postoperative period in this study (28). A limited number of studies have investigated the time point at which the IGF-I level stabilizes after pituitary surgery. Patients’ IGF-I levels can become normalized early after surgery (within weeks), but delayed stabilization of up to 12 months can also occur (29–31), which may partly explain why fewer patients achieved a normal age-adjusted IGF-I level (33.5%) than GHn level (56.9%) (Table 2). Previous research (12) has shown that discordance between GH and IGF-I levels can occur in up to 30% of patients with acromegaly after treatment, and most discordance involves normal GH levels and elevated IGF-I levels. This discordance may arise due to many factors such as pulsatility, age, comorbidities, and genetic differences (12), including the longer half-life of the IGF-I hormone, which might result in a higher IGF-I level in the short time between surgery and the OGTT test. Another possible reason for this discordance is that very subtle abnormalities of GH secretion can be sufficient to increase IGF-I production into a supranormal range (32). In acromegaly, the tumor produces GH but not IGF-1, and absolute IGF-1 levels or IGF-1 z-scores increase nonlinearly with GH levels, which results in a far greater extent of nonlinearity between GH and IGF-1 levels than has been previously recognized (33). However, the exact mechanism of this phenomenon remains unclear, and revealing it will require the measurement of IGF-1 and GH levels to monitor tumor activity.

In our study, a lower follow-up %ULN IGF-I was associated with an improved glucose status at an average of 133.76 days after surgery, indicating that the IGF-I level at approximately 4 months after surgery was already predictive of the glucose status. These findings indicate the importance of the early measurement of IGF-I in patients with an abnormal blood glucose level. These results were further confirmed by comparisons of the glucose levels according to the IGF-I criterion of a cure (Table 4). The follow-up glucose levels of the cure group were lower than were those of the discordant group after surgery.

Furthermore, to confirm the correlation between the %ULN IGF-I and glucose levels before and after surgery, Pearson's correlation analysis was performed. The correlations detected between the laboratory indexes indicate that the change in %ULN IGF-I between before and after surgery might be predictive of an altered glucose level, despite the weak correlation observed. However, no significant relationships were found between the differences in the random GH or GHn levels before versus after surgery and the glucose parameters. Thus, the IGF-I level was a better indicator than the GH-related indexes of altered glucose metabolism in the acromegalic patients.

This conclusion is in agreement with those of previous studies reporting a closer correlation of IGF-I with indexes of insulin resistance than nadir or random GH (8–10). Hopkins and cols. first reported that IGF-I, but not GH, was significantly correlated with insulin resistance; however, the sample examined was small, including only ten patients with active acromegaly, seven with controlled disease and 22 normal individuals (9). Another study investigated 92 Japanese acromegalic patients who underwent surgery, but only a weak correlation between the IGF-I Z score and HOMA2-S % before surgery was reported; no post-op data were reported (8). Dan and cols. also reported that IGF-I was associated with glucose intolerance, but they assessed only 29 patients treated with surgery without considering any preoperative parameters (10). Our study, which involves the largest patient sample (218 patients) with complete follow-up data, is the largest study to correlate %ULN IGF-I with the glucose tolerance status both before and after surgery.

The specific physiological and pathological mechanisms underlying the correlation of IGF-I with glucose status in acromegaly is still unclear. However, the measurement of serum IGF-I may be the best single test for the diagnosis of acromegaly and may reflect the activity of the disease, as serum IGF-I concentrations do not vary according to daily activities but instead reflect integrated GH secretion during the preceding day or longer (6,34). This result may partially explain the relationship between %ULN IGF-I and glucose status observed in our study. Considering our findings, further study of the specific physiological and molecular mechanisms of the effects of IGF-I on glucose metabolism is warranted.

These observations suggest that the age- and sex-adjusted IGF-I level before and after TSA and its change are associated with improved glucose tolerance in acromegalic patients both before and after TSA. We suggest that the continuous monitoring of the serum IGF-I level is necessary to evaluate blood glucose improvement in these patients. For better long-term prognosis, improved control of blood glucose should be one of the goals in the management of acromegaly. Considering this goal, we advise placing more emphasis on the IGF-I level at 3 months after surgery. A lower level indicates better recovery and control of DM and the IGF level; thus, more accurate criteria for the cure and control of acromegaly as well as guidelines that consider the treatment of complications must be established.

### Limitations

There are several limitations in our study. Our study was designed as a retrospective study with a relatively short follow-up period; thus, a long-term association of IGF-I with glucose metabolism could not be examined. Moreover, with incomplete data, we failed to assess other markers for glucose metabolism before and after surgery, such as the HbA1c level, serum insulin levels and insulin sensitivity. As ours was a single-center study, additional multi-center studies should be performed.

In conclusion our study of acromegalic patients is the first to report complete preoperative and follow-up data. The results suggest that the age- and sex-adjusted IGF-I level is an effective parameter that reflects changes in glucose metabolism and that is associated with improved glucose tolerance in acromegalic patients both before and after TSA. Careful monitoring of the serum IGF-I level is recommended to evaluate blood glucose improvement in acromegalic patients.

## Supplementary data

Pearson's correlation matrix for laboratory parameters before and after TSA

**Table:**

	%ULN (B)	%ULN (F)	Δ%ULN	GH (B)	GH (F)	ΔGH	GHn (B)	GHn (F)	ΔGHn	Glu0 (B)	Glu0 (F)	ΔGlu0	Glu120 (B)	Glu120 (F)	ΔGlu120
%ULN (B)	1	0.320**	-0.694**	0.193**	0.007	-0.200**	0.184**	0.029	-0.188**	0.180**	0.089	-0.174*	0.231**	0.157*	-0.153*
%ULN (F)		1	0.460**	0.339**	0.483**	-0.253**	0.328**	0.583**	-0.249**	0.046	0.198**	0.075	-0.013	0.168*	0.202**
Δ%ULN			1	0.077	0.361**	-0.005	0.077	0.415**	-0.013	-0.134*	0.067	0.220**	-0.227**	-0.019	0.297**
GH (B)				1	0.308**	-0.980**	0.974**	0.326**	-0.966**	0.067	-0.020	-0.101	0.013	-0.055	-0.078
GH					1	-0.112	0.338**	0.942**	-0.201**	0.052	0.090	-0.006	0.031	0.018	-0.023
ΔGH						1	-0.946**	-0.144*	0.966**	-0.059	0.040	0.104	-0.007	0.061	0.076
GHn (B)							1	0.354**	-0.988**	0.063	-0.043	-0.112	-0.002	-0.068	-0.070
GHn								1	-0.208**	0.035	0.105	0.027	0.002	0.050	0.052
ΔGHn (F)									1	-0.060	0.062	0.121	0.003	0.079	0.082
Glu0 (B)										1	0.650**	-0.854**	0.798**	0.637**	-0.427**
Glu0 (F)											1	-0.161*	0.624**	0.710**	-0.103
ΔGlu0												1	-0.610**	-0.342**	0.485**
Glu120 (B)													1	0.706**	-0.635**
Glu120 (F)														1	0.099
ΔGlu120															1

** significantly correlated at the 0.01 level (two sides);

* significantly correlated at the 0.05 level (two sides); (B): at baseline; (F): at follow-up; Δ: change from baseline; Glu0h: basal glucose level on OGTT; Glu120: 120-min glucose level on OGTT; GH: growth hormone; GHn: GH nadir; IGF-I: insulin-like growth factor-I; ULN: upper limit of normal range.
